# A passive wireless surface acoustic wave (SAW) sensor system for detecting warfare agents based on fluoroalcohol polysiloxane film

**DOI:** 10.1038/s41378-023-00627-8

**Published:** 2024-01-03

**Authors:** Yong Pan, Cancan Yan, Xu Gao, Junchao Yang, Tengxiao Guo, Lin Zhang, Wen Wang

**Affiliations:** 1 State Key Laboratory of NBC Protection for Civil, Beijing, 102205 China; 2 Institute of Acoustics, Chinese Academy of Sciences, Beijing, 100190 China; 3 The School of Electronic, Electrical and Communication Engineering, University of Chinese Academy of Sciences, Beijing, 100049 China

**Keywords:** Carbon nanotubes and fullerenes, Chemistry

## Abstract

Long-term monitoring of environmental warfare agengts is a challenge for chemical gas sensors. To address this issue, we developed a 433 MHz passive wireless surface acoustic wave (WSAW) gas sensor for dimethyl methylphosphonate (DMMP) detection. This WSAW gas sensor includes a YZ lithium niobate (LiNbO_3_) substrate with metallic interdigital transducers (IDTs) etched on it, and an antenna was placed near the IDT. A DMMP-sensitive viscoelastic polymer fluoroalcoholpolysiloxane (SXFA) film was prepared on a LiNbO_3_ substrate, and mode modeling coupling was used to optimize the design parameters. The sensor can function properly in an environments between −30 °C and 100 °C with humidity less than 60% RH. When the wireless transmission distance was within the range of 0–90 cm, the sensor noise increased with distance, and the stability was less than 32°/h. While optimizing the film thickness of SXFA, a relationship was observed between sensor sensitivity and film thickness. When the film thickness of SXFA reached 450 nm, the optimal value was reached. At a distance of 20 cm between the transmitting and receiving antennas, DMMP was detected at different concentrations with the developed WSAW gas sensor. The lower detection limit of DMMP was 0.48 mg/m^3^, the sensitivity of the sensor was 4.63°/(mg/m^3^), and repeatable performance of the sensor was confirmed.

## Introduction

As chemical warfare agents (CWAs) threaten the security of the world, the development of a gas sensor with features such as fast response, high sensitivity, and small size is imperative for use in early detection of CWAs. Simultaneously, accurate detection, identification, and monitoring of CWAs is essential for effective military and civilian defense operations. Because CWAs are highly toxic and deleterious, for security reasons, they are only studied in specific places, which are limited to authorized laboratories. Therefore, their simulants, which can closely mimic the chemical structures of real CWAs without associated toxicological properties^1–4^, have often been studied in place of real CWAs.

Among the various types of CWAs, the nerve agent sarin (O-isopropyl methylphosphonofluoridate, GB) is the most lethal because of its high toxicity and moderate volatility. It can cause death at a very low concentration. Thus, dimethyl methylphosphonate (DMMP) is often used as a GB simulant, not only because its polar (*P* = O-, P-O-) and nonpolar (-CH_3_) functional groups are the same as sarin but also because of its nontoxic physicochemical property during testing experiments^5–8^.

Surface acoustic wave (SAW)-based sensors have attracted substantial interest as sensing platforms to detect physical, chemical, and biological substances^9–11^. Because SAWs have exhibited many unique advantages, such as fast response, ambient-temperature operation, high sensitivity, low cost, easy reproducibility, and favorable stability^12–16^, it has been widely used in detecting CWAs^17–22^ in recent years. However, for all CWA alarms powered by batteries, problems such as short duration, large power consumption in a low-temperature environment, and the impact of battery replacement during detection have not been solved. To address this situation, we coupled passive wireless surface acoustic wave (WSAW) gas sensing; this technology ran on battery power and could also perform CWA detection through radar transmission. The development of WSAW sensors has been reviewed in recent years^23–27^. An increasing number of passive wireless sensors have been used in physical parameter detection of temperature^28–31^, pressure^32–34^, and moisture^35,36^. Passive SAW-based sensors for wirelessly sensing harmful gases, such as CO_2_^37–43^, NO_2_^44,45^, NH_3_^46^, O_2_^47,48^, and other volatile organic compounds, were proposed, where the phase signal from the reflectors of the SAW reflective delay line configuration induced by gas adsorption between the sensitive interface and target gas was picked for gas sensing^49^. All these studies provide favorablesuitable references for developing a real practical wireless and passive SAW chemical sensor.

**Fig. 1:**
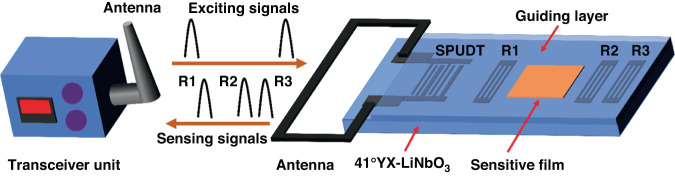
Schematic and working principle of the proposed SAW chemical sensor

In this study, we developed a new passive WSAW chemical sensor with a 433 MHz central frequency and an associated viscoelastic sensitive layer fluoroalcohol polysiloxane (SXFA) compatible with the transducer, which enabled detection of DMMP. The schematic and working principle of the sensor are illustrated in Fig. 1. The developed platform consists of a SAW reflective delay line, reader unit, and connected antennas. In the reflective delay line, single-phase unidirectional transducers (SPUDTs) and reflectors were placed in a row along the SAW propagation direction on a YZ LiNbO_3_ piezoelectric substrate^39^. When the SPUDTs received electromagnetic (EM) energy from the reader unit through a connected antenna, the SPUDTs generated a SAW on the surface through the piezoelectric effect and propagated it toward the reflectors. The propagating SAW was partially reflected from the reflectors, and the reflected SAW was reconverted into EM waves by the SPUDTs and transmitted to a reader unit. Then, the adsorption of DMMP on the SXFA polymer film can induce a change in mass loading and SAW velocity, which results in a phase shift. By evaluating the phase shifts, we extractd the gas concentration.

Compared with the currently available gas sensors, the developed passive WSAW sensor has many advantages. First, batteries or any power supply was not needed to operate the sensor. Second, the sensor can be used in hazardous areas that are not easily accessible to personnel, and the sensor can be operated wirelessly. Third, it can be operated without a complicated detection system. Finally, the sensor was lightweight, small, and could withstand extremely harsh environmental conditions.

## SAW device design and fabrication

### Design considerations of reader unit

**Fig. 2:**
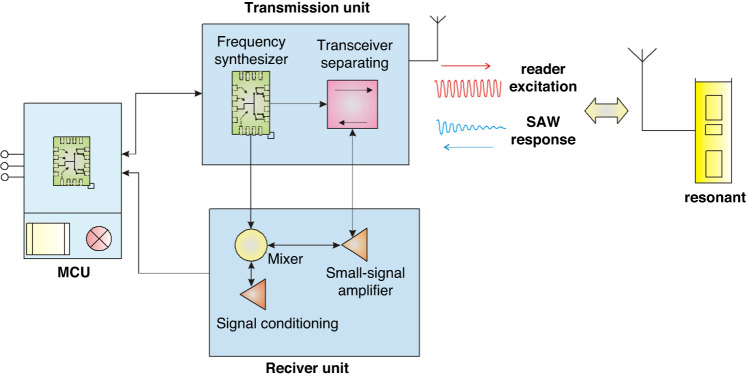
Structure of the FSCW reader unit used

A hardware structure for the reader includes the main control unit (MCU), transmitting unit, and receiving unit, as shown in Fig. 2. A frequency-stepped continuous wave radar technique, which can generate a continuously variable frequency sine wave excitation signal, was used in our research^50^. The arrow direction in Fig. 2 indicates the propagation direction of the signal, transmitting unit, and receiving unit that share a transceiver antenna. The MCU communicated with the PC through 3-wire UART, the antenna, and the SAW sensor exchange through EM waves.

The MCU comprised an STM32 single-chip microcomputer and its auxiliary circuit. STM32 is a powerful embedded microprocessor chip that is powerful for computing and integrated many peripheral units. This unit also included radar control, digital-to- analog conversion, display, and communication interface functions.

The transmitting unit comprised a frequency synthesizer and a transceiver separating circuit. The frequency synthesizer was responsible for providing signals for the local oscillator end of the transmission circuit and the mixing circuit. The main function of the frequency synthesizer was to generate sine waves of different frequencies and simultaneously ensure that the power of the signal provided would meet the requirements of the subsequent parts. The transceiver separation circuit was responsible for the separation of high-frequency transmitted signals and received signals so that they could move forward according to the predetermined signal transmission without mutual interference or conflict.

The receiving unit comprised a small signal amplifier, a frequency mixer, and a signal conditioning component. The small signal amplifier is responsible for preliminary amplification of the received reflected signal to prevent excessive noise in the circuit from obfuscating the useful received signal and to simultaneously prevent introducing excessive noise. The frequency mixer is responsible for mixing the received signal with the local oscillator signal to reduce the received signal to the low-frequency band that can be sampled. The signal conditioning part is responsible for amplifying and shaping the low-frequency signal after mixing to ensure that the signal meets the requirements of the subsequent sampling circuit and does not affect the information carried in the signal.

### Design and fabrication of the SAW sensor

**Fig. 3:**
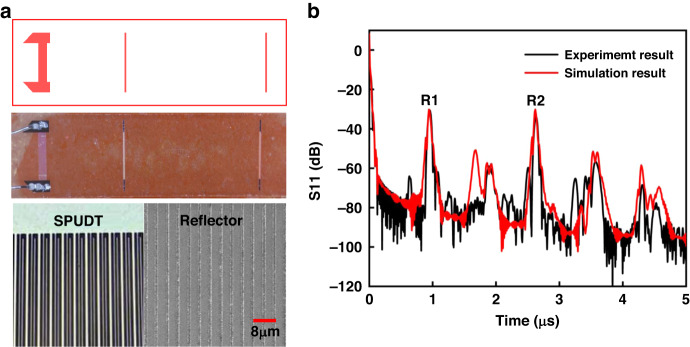
**a** Prepared physical diagram of the delay line device. **b** Comparison between experimentally measured and simulated reflection peaks in the time domain

A reflective delay line structure was used as a SAW sensor. To obtain sensor devices with favorable performance, we consider designs that improve the signal-to-noise ratio (S/N) and reduce the insertion loss, as well as factors influencing sharp reflection peaks in the time domain and spurious noise^51^. Thus, the YZ LiNbO_3_ piezoe-lectric crystal was selected as the sensor substrate due to its large electromechanical coupling Factor K_2_ (4.5%). SPUDTs were used in the fabricated SAW sensor because they could direct most of the SAW energy to the reflectors, and thus, high S/N and lower insertion loss were ensured^52^. The operation frequency of the SAW sensor was set to 433 MHz to meet the requirement of the sweep frequency bandwidth of the reader unit. The SAW sensor used for sensing gas was simulated to obtain S11 in the time domain. Table 1 shows the design parameters, and Fig. 3 shows that the relative position of the reflection peak is consistent with that of the sensor design, and a high S/N is obtained.

**Table 1:** SAW sensor design parameters

Structure parameters	Value
Piezoelectric	YZ-LiNbO_3_
Acoustic wavelength	8 μm
IDT pairs	25
Electrode	Al
Reflector electrodes	10
Type of reflectors	short-circuited gratings
Aperture	100λ

### Optimal selection of devices with different structures

The stability of sensor devices is crucial, as it directly affects the detection effect of the sensor on the target gas. To obtain SAW sensor devices with favorable performance in simulation, six types of SAW devices with different structures are designed and manufactured, as shown in Table 2.

**Table 2:** Structure and peaks (reflectors 1 and 2) of SAW devices

SAW device structure				Peak value (dB)		
Device type	IDT structure	Position of reflectors	Metal thin film	Reflector 1	Reflector 2	S/N
1	IDT	Right side of IDT	/	−28.0	−29.7	52
2	SPUDT	Right side of IDT	/	−45.9	−47.9	35
3	IDT	Right side of IDT	Al film	−29.0	−32.3	48
4	SPUDT	Right side of IDT	Al film	−40.1	−43.3	40
5	IDT	Both sides of IDT	Al film	−28.4	−30.6	50
6	IDT	Both sides of IDT	/	−27.7	−29.4	53

For types 1 and 2 devices, the reflection peak S/N of the bidirectional acoustic transmission IDT structure was approximately 10 dB higher than that of the SPUDT structure device. More narrowly, the prepared SPUDT structure device had more line defects due to the limitation of the production process level, which results in poor device performance, such that it was not used. For types 3, 4, and 5 devices, the difference between the devices with and without aluminum film, that is, the second reflection peak, was approximately 3–5 dB smaller than the first because of the acoustic attenuation caused by the discontinuity of the aluminum film boundary. The subsequent coating of the sensitive film also led to acoustic attenuation. The greater the thickness is, the greater the acoustic attenuation, and the lower the S/N, so a decrease in the S/N ratio affects the performance of the sensor. For type 6 devices, the second reflection peak was approximately 0.5 dB because of the high utilization of acoustic waves and high S/N of the two-sided reflector structure compared with the single-sided reflector structure.

According to the S/N, loss, and ability of the device to withstand film thickness during the later coating process, type 6 (bidirectional acoustic propagation IDT + , bilateral reflectors + aluminum film free structure) devices have higher S/Ns and peak values, which make them more suitable as sensor structures for detecting gases, thereby achieving the maximum sensitivity of the prepared sensor.

### Measurement setup

**Fig. 4:**
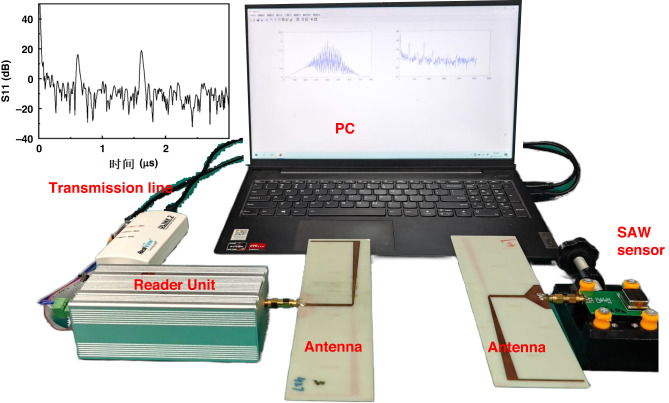
Wireless measurement setup of the SAW gas sensor system

Figure 4 shows the experimental setup used for sensing data collection. Two fabricated antennas were connected to the reader unit and fabricated microsensor, and the reader unit was connected to the PC with transmission lines. The working process of the entire system was as follows: the EM wave signal emitted by the reader was transmitted to the sensor through the antenna and then converted into a SAW signal by an interdigital transducer. This SAW signal propagated along the substrate to the reflector and was reflected back and then converted back into an electrical signal through an interdigital transducer, with the reader receiving the signal through the antenna.

### Temperature and humidity experiments for the WSAW sensor system

**Fig. 5:**
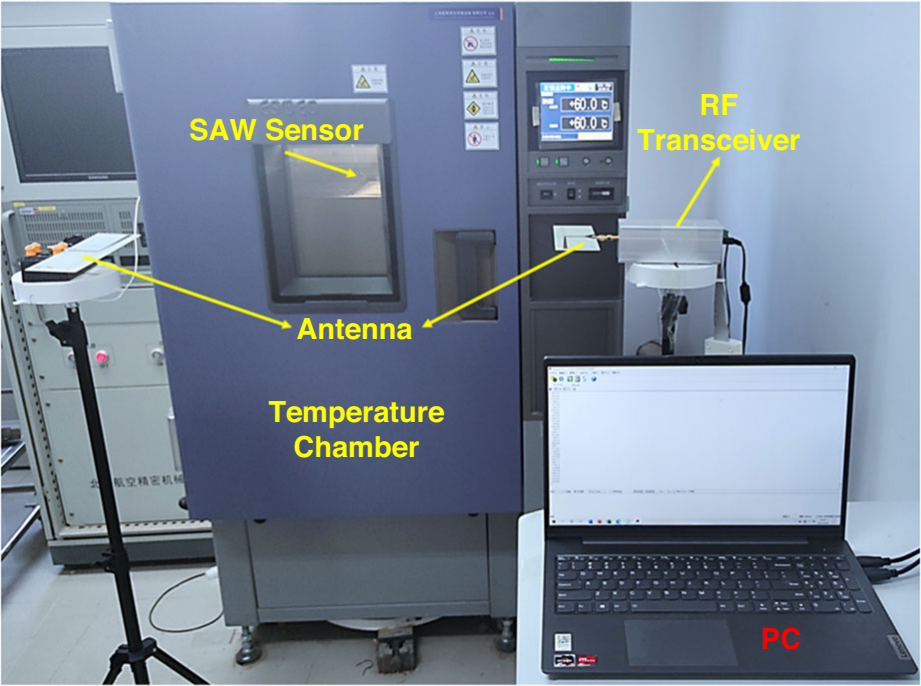
WSAW sensor detection system for the temperature test

Temperature and humidity experiments were carried out to further verify the environmental adaptability of the test system. This temperature test setup is illustrated in Fig. 5.

**Fig. 6:**
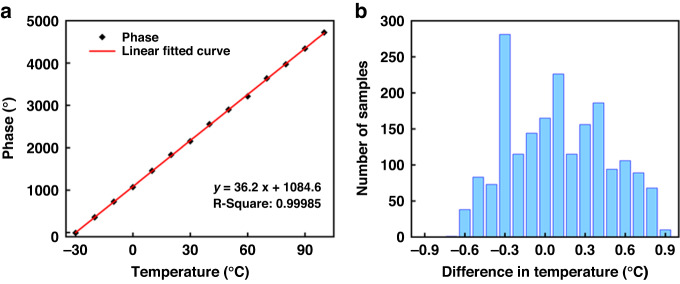
**a** System temperature test and (**b**) temperature test error diagram

Within the range of −30–100 °C, a temperature measurement point was set at every 10 °C interval, and 150 sets of tests were conducted at each temperature measurement point. The test results are shown in Fig. 6. The temperature measurement sensitivity was approximately 36.2 °C, and the temperature measurement accuracy reached ±0.5 °C. The results showed that the test platform worked well within the temperature range of −30–100 °C.

**Fig. 7:**
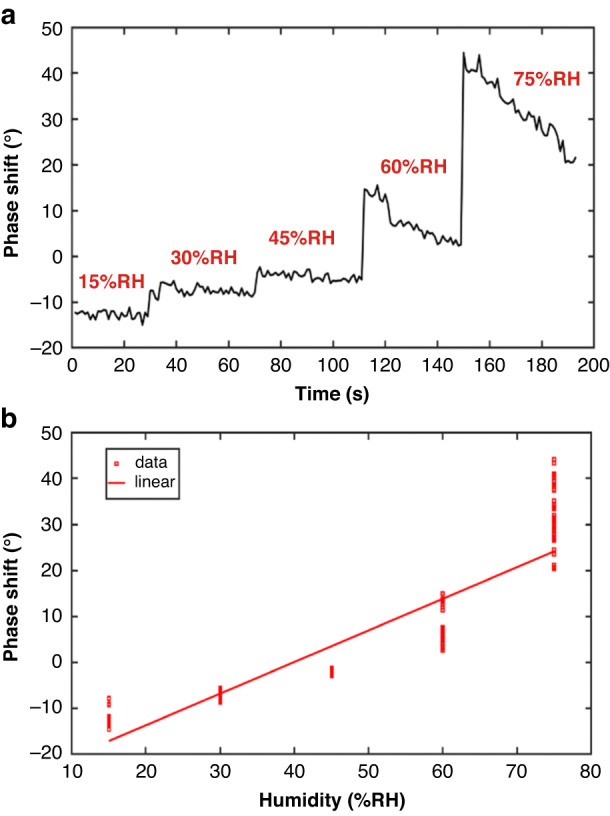
**a** System humidity test and (**b**) humidity detection error diagram (21 °C, 30% RH)

The humidity experiment was conducted with a distance of 15 cm between the transmitting and receiving antennas, and the tests were conducted on five humidity points with relative humidities of 15%, 30%, 45%, 60%, and 75% RH. The results are shown in Fig. 7a. When the humidity was less than 60% RH, the phase change became larger as the humidity increased, with a sensitivity of approximately 1°/% RH. However, when the humidity reached 60% RH and 75% RH, a large fluctuation occurred in the sensor baseline. As shown in Fig. 7b, the error of the system is large, and the accuracy is low, indicating that when the ambient humidity is greater than 60% RH, the stability of the sensor can be affected.

## Results and discussion

### Experimental section

**Fig. 8:**
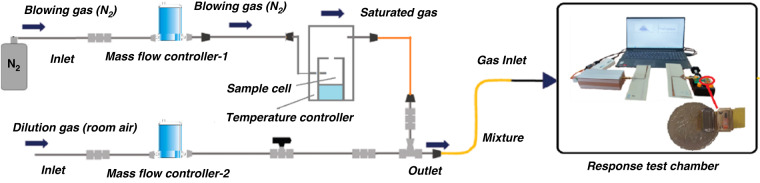
Schematic diagram of the instrument and setup used for data acquisition and management

To prepare the WSAW gas sensor, SXFA was prepared according to ref. ^8^, and SXFA with a concentration of 9.5 mg/ml was prepared in benzene and spun onto a quartz wafer with delay line patterns. The spinning speed and duration were set at 1200 rpm and 10 s, respectively. Then, the WSAW sensor could be obtained after standing, high-temperature aging, cutting, and packaging. To obtain realistic data, a new gas generating system was established by our laboratory, as shown in Fig. 8. In this setup, ambient air was used as the dilution and recovery gas, and DMMP was placed in a sample cell of the homemade gas generation system. When DMMP was blown with N_2_ and diluted with ambient air, a given concentration of DMMP was formed, the mixture gas concentration was monitored in real time using a gas concentration monitor, which was developed by our laboratory, and the prepared WSAW sensor was placed in a response test chamber with a gas inlet and outlet. When the generated gases with different concentrations have directly entered the chamber, the response signals of the sensor toward DMMP were recorded and collected in real time by a PC. All testing was conducted at room temperature.

### Relationship between the wireless transmission distance and baseline noise

For WSAW gas sensors, during wireless signal transmission, the S/N was not only affected by uncontrollable environmental factors but also decreased exponentially as the wireless distance increased, as shown by Eq. (1).

1 $${\rm{Loss}}=20\mathrm{ln}\frac{4\pi R}{{\lambda }}=32.45+20{\rm{lnf}}({\rm{MHz}})+20\mathrm{ln}{\rm{R}}({\rm{km}})$$

where Loss is the transmission loss (dB), R is the transmission distance (km), and f is the frequency (MHz). When the sensor was coated with a sensitive polymer film between the first and second reflectors, the phase shift ∆∅ between the two reflections because of gas adsorption was obtained by the fractional change in the propagation velocity in Eq. (2).

2 $$\Delta {{\varnothing }}\,=\,2{{\pi }}{\rm{f}}_0\Delta {{\tau }}=2{{\pi }}{\rm{f}}_0\times \,2{\rm{l}}_1/{\rm{v}}_0\,\times \left(\frac{{\Delta}_v}{{v}_{o}}\right)$$

where f_0_ is the central frequency, l_1_ is the distance between the first and second reflectors, ∆τ is the time delay change, v_0_ is the acoustic velocity, and $\frac{{\Delta}_v}{{v}_{o}}$ is the fractional change in propagation velocity.

**Fig. 9:**
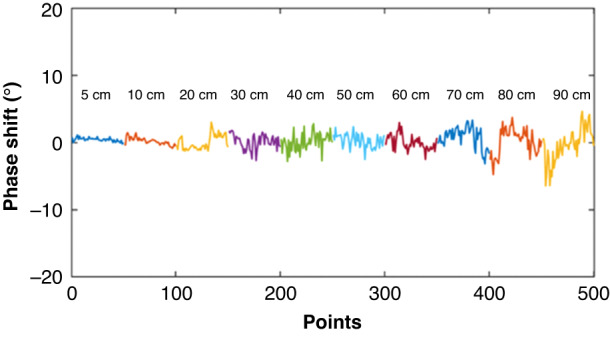
Baseline noise value at different wireless distances (25 °C, 40% RH)

Simultaneously, the baseline noise of the WSAW sensors is affected by environmental factors. The stability tests were conducted with distances between the transmitting and receiving antennas of the WSAW sensor within a range of 5, 10, 20, 30, 40, 50, 60, 70, 80, and 90 cm. Figure 9 and Table 3 show the changes in baseline noise of the WSAW sensor at different distances.

**Table 3:** Changes in baseline noise over different wireless distances

Distance (cm)	5	10	20	30	40	50	60	70	80	90
Phase shift (°)	1.35	2.33	4.30	4.51	4.85	5.19	5.50	6.52	8.50	11.63

**Fig. 10:**
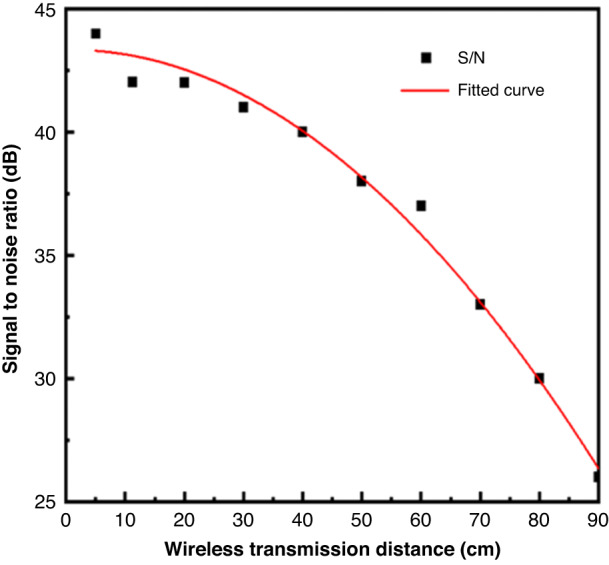
S/N under different wireless distances (21 °C, 40% RH)

These results showed that the shorter the distance between the transmitting and receiving antennas, the more stable the baseline noise would be, and the higher the S/N. The longer the distance is, the greater the baseline noise, and the lower the S/N, as shown in Fig. 10. Considering factors such as sensor noise, sensitivity, and environmental impact, we applied a wireless transmission distance of 20 cm for additional testing.

### Stability of the WSAW sensor

**Fig. 11:**
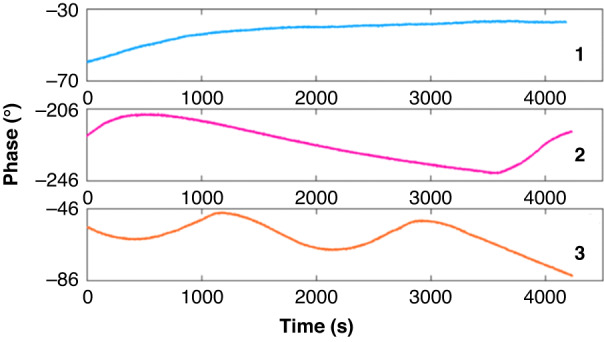
Stability of the WSAW sensor (23 °C, 38% RH)

As discussed above, when the distance between the transmitting and receiving antennas is less than 30 cm, the WASW might be stable. Three devices were selected to confirm the stability of the prepared WSAW sensors, and the distance was set to 20 cm. We found that the phase shift was approximately 25°/h, 32°/h, and 35°/h, which indicated that the sensor could achieve long-term stability at this distance, as shown in Fig. 11.

### Relationship between WSAW sensor response signal and baseline noise

**Fig. 12:**
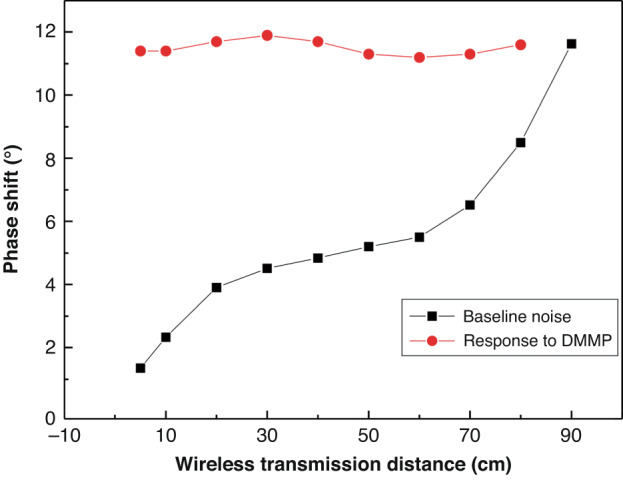
Relationship between WSAW sensor baseline noise and response to DMMP (21 °C, 35% RH)

DMMP with a concentration of 3.8 mg/m^3^ was tested using the prepared sensors at different wireless transmission distances, as shown in Fig. 12. The figure shows the baseline noise fluctuation value and response value to DMMP detection at various wireless distances. As the distance increased, the baseline noise fluctuation of the WSAW sensor gradually increased, but the response values of the WSAW sensor to DMMP at different wireless distances were basically consistent and did not change. This result indicated that the response signal of the WSAW sensor to a certain concentration of target gas was independent of the wireless distance, but as the transmission distance increased, the baseline noise gradually increased, ultimately resulting in an S/N that was too small to enable accurate detection.

### Relationship between the sensor response and SXFA film thickness

As a type of glassy-rubbery polymer material, the characteristic functional group -CF_3_ in SXFA has hydrogen bond acidity and can selectively adsorb DMMP, an organic phosphorus compound with hydrogen bond alkalinity at room temperature. This condition mainly occurs due to the reversible hydrogen bonds that are formed between weak acid and weak base functional groups. The main effects of polymer films on the propagation characteristics of SAW are viscoelastic effects and mass loading effects, as reported in Eq. (3).

3 $$\frac{{\Delta }_{{\rm{Y}}}}{{{\rm{K}}}_{{\rm{o}}}}=\frac{{\Delta }_{{\rm{\alpha }}}}{{{\rm{K}}}_{{\rm{o}}}}-{\rm{j}}\frac{{\Delta }_{v}}{{v}_{{\rm{o}}}}=\mathop{\sum}\limits_3^{\rm{i}}=1\frac{{{\rm{c}}}_{{\rm{i}}}{{\rm{\beta }}}_{{\rm{i}}}{{\rm{M}}}^{\left({\rm{i}}\right)}}{{\rm{\omega }}}\tanh \left({\rm{j}}{{\rm{\beta }}}_{{\rm{i}}}{\rm{h}}\right)$$

In Equation (3), ${\beta }_{i}^{2}={\omega }^{2}(\rho -{E}^{i}/({V}_{0}^{2}))/{M}^{(i)}$, ${\rm{V}}_{0}$ is the SAW velocity after disturbance, and E_i_ is the deformation modulus, E^1^ = G, E^2^ = 0, E^3^ = 4 G(3 K + G)/(3 K + 4 G), M^1^ = M^3^ = G, M^2^ = K, where G and K are the shear modulus and bulk modulus of the polymer, respectively, and are usually complex quantities.

**Fig. 13:**
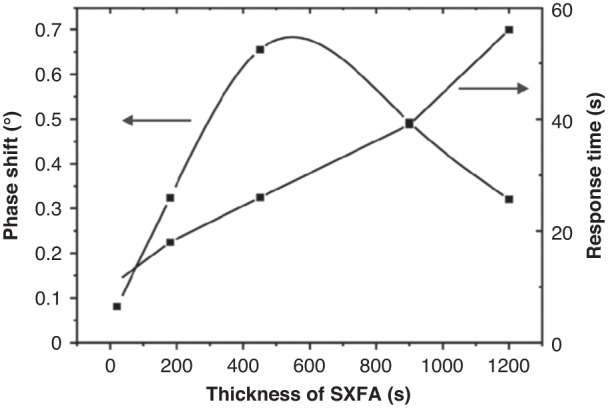
Relationship between the sensor response and SXFA film thickness (22 °C, 35% RH)

SXFA sensitive films with different thicknesses were prepared by the spin method. The SXFA toluene solution with a concentration of 10 mg/mL was prepared, and the spinning speed and duration were set to 1500 rpm and 15 s, respectively. The SXFA toluene solution was spun onto the Y-cut quartz wafer with delay-line patterns to form the gas sensor. Then, the SAW sensor was obtained using high-temperature aging, cutting, and packaging after standing. When the concentration of the measured gas DMMP was 2 mg/m^3^, and the distance between the transmitting and receiving antennas was 20 cm. The relationship between the response value of the SAW sensor and the film thickness is shown in Fig. 13.

As shown in Fig. 13, no monotonically increasing change is observed between the sensor response signal and the film thickness, but an optimal thickness is found because of the viscoelastic effect of SXFA as a polymer-sensitive material. As SXFA used in this manuscript is a kind of multiple molecular layer, according to linear solvation energy relations (LSERs) and gas‒liquid equilibrium theory^7,8,53^, at a certain temperature, the ratio of the concentration of molecules in the gas phase C_v_ to the concentration on the membrane surface C_s_ is a constant K. When the concentration of molecules in the gas phase is constant, under its pressure drive, if the membrane is very thin, it will quickly reach equilibrium. If the membrane is too thick, the permeation of gas molecules in it leads to a longer equilibrium time. Therefore, the film must be prepared based on an optimal thickness. In addition, the response time of the sensor also increases with increasing membrane thickness, which is due to the gas permeation effect between multilayer membrane materials. The thicker the membrane is, the longer it takes for the gas to resolve from the adsorption surface. Therefore, the optimized SXFA film thickness of approximately 450 nm is suitable.

### Polarizing microscopy analysis of the SXFA Film

**Fig. 14:**
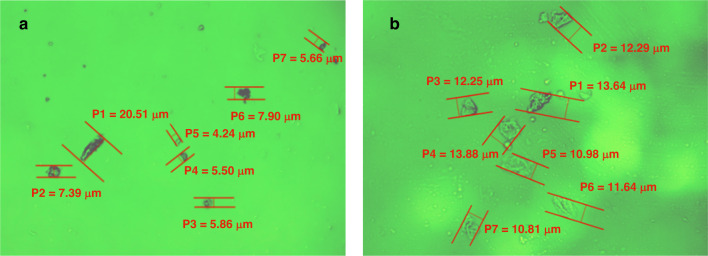
Polarizing microscopy analysis of SXFA films. **a** and **b** represent device a and device b, respectively

The performance consistency of gas sensors has always been an important aspect of sensor research. Although the same preparation methods have been used in the preparation process of sensors, the surface morphology of the obtained sensor material and its response to the measured gas are not completely consistent, and sometimes significant differences occur. Using spin coating method, SXFA was applied over the SAW delay line. By selecting two sensors, which are marked by device (a) and device (b), we found that the majority of the film area was uniform under the polarizing microscope, which is shown by the green transparent region of Fig. 14. However, as a kind of viscoelastic material, cluster particles will inevitably form during high-temperature aging because of the evaporation of solvent. In the range of 200 μm × 200 μm, seven cluster particles in a region with more clusters were selected to analyze the surface uniformity separately, and the particle sizes of various clusters are shown in Table 4 and Fig. 14.

**Table 4:** Particle sizes of different SAW-SXFA films obtained using polarizing microscopy

Device	P1 (μm)	P2 (μm)	P3 (μm)	P4 (μm)	P5 (μm)	P6 (μm)	P7 (μm)	Average (μm)
a	20.51	7.39	5.86	5.50	4.24	7.90	5.66	8.15
b	13.64	12.29	12.25	13.88	10.98	11.64	10.81	12.21

Table 4 shows that the selected cluster shapes are random. The seven cluster diameters in (a) or (b) in Fig. 14 are basically similar: the average diameters of cluster particles in device (a) and device (b) are 8.15 μm and 12.21 μm, respectively, most clusters have diameters of approximately 10 μm, and the particle diameters between devices (a) and (b) are also substantially the same.

At the same time, the areas beyond the 14 clusters are transparent, indicating the absence of clustering in these areas because SXFA is a transparent gel-like polymer. The results show that a uniformly sensitive film with favorable performance can be obtained by controlling the spin coating conditions.

### Atomic Force Microscopy (AFM) interface analysis of the SXFA Film

**Fig. 15:**
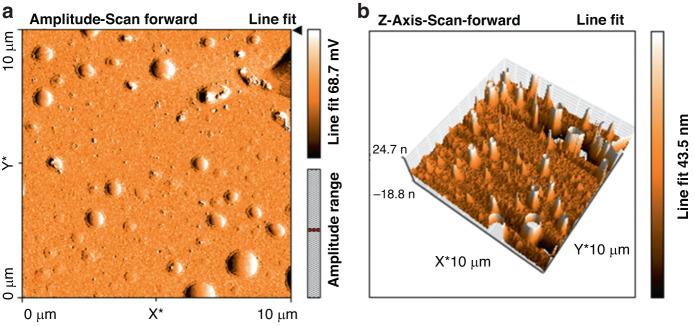
Analysis of the SXFA film by AFM. **a** 2D and (**b**) 3D spectra of SXFA

For imaging of a 10 μm × 10 μm area, interface analysis of the surface of the SXFA film was performed using atomic force microscopy (AFM) within the image range to further obtain more relevant surface information (Fig. 15 and Table 5). In the 2D photograph in Fig. 15a, light colors correspond to upward bumps in the film, while dark colors correspond to downward depressions. They are considered uniform because of their low color contrast. The 3D photo shown in Fig. 15b shows the height difference between the darkest and brightest parts of the uneven structure. As the dark part is clearly more uniform and regular than the bright part, the surface of the surface facial mask is considered uniform. This result is further confirmed by cluster particle state of the polarization micrograph.

**Table 5:** Roughness analysis of the SXFA film surface by AFM

Area	Sa	Sq	Sy	Sp	Sv	Sm
100.8 pm^2	5.4797 nm	10.786 nm	155.44 nm	123.37 nm	−32.079 nm	−20.125 fm

Generally, when the surface roughness Sq of the film is less than 20 nm, the surface of the film is considered uniform. Table 5 shows that the S_q_ value of the SXFA film is 10.786 nm, indicating that the prepared SXFA film is uniform, as shown in Table 5.

### SEM interface analysis of the SXFA film

**Fig. 16:**
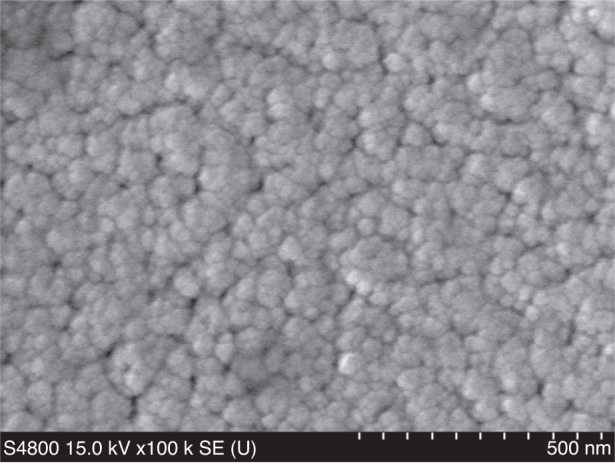
SEM image of the SXFA film on the SAW delay line

To verify the morphology and surface coverage of SXFA thin films and confirm their surface morphology, we performed SEM for surface analysis of the films coated on the SAW delay line. Figure 16 shows an enlarged cross-sectional view of the film. The image shows that the grown film is porous and contains particle morphology throughout the substrate. After spin coating and solvent volatilization, the specific surface area of the film increases, indicating that the microstructure is conducive to 3D adsorption of the measured gas. Figure 16 also shows that the polymer has an irregular geometric shape. Therefore, the prepared SXFA film is an amorphous polymer.

### WSAW gas sensor performance

**Fig. 17:**
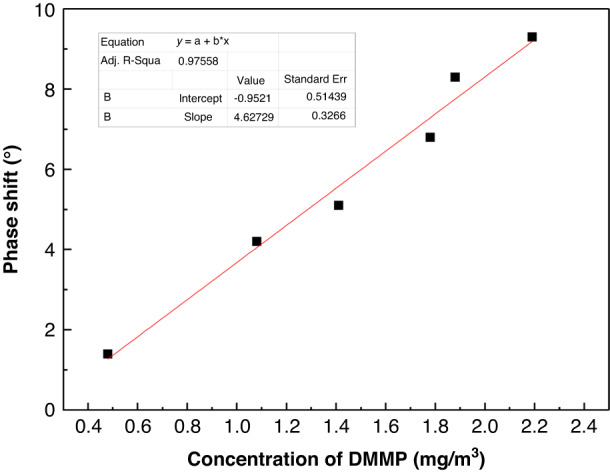
Relationship between the WSAW sensor response signal and DMMP concentration (21 °C, 33% RH)

**Fig. 18:**
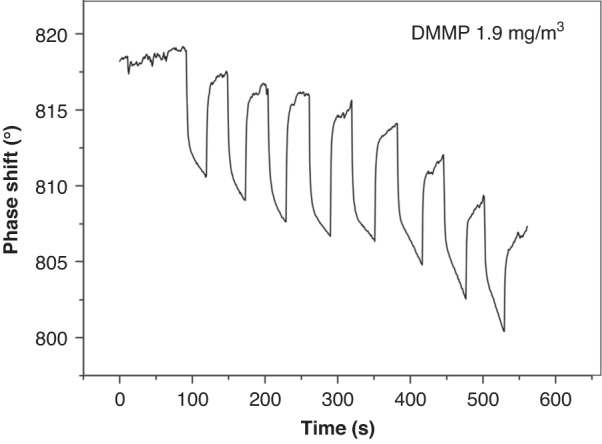
Curve indicating eight consecutive detections of DMMP (25 °C, 35% RH)

At room temperature, we set the distance between the transmitting antenna and receiving antenna to 20 cm and used the gas dynamic generation method to obtain various concentrations of DMMP gas^53^. Then, the DMMP was detected using the developed WSAW sensors. The linear relationship and repeatability are shown in Figs. 17 and 18.

In Fig. 17, a favorable linear relationship is shown between the detection concentration and response signal, y = −0.95212 + 4.63x, *R* = 0.97558, and the sensitivity of the sensor is 4.63°/(mg/m^3^). When the DMMP concentration increases, the response signal of the SXFA sensor also increases, with a minimum detection concentration of 0.48 mg/m^3^. This curve indicates that a specific and selective interaction relationship can be formed between the gas and polymer at low concentrations. This interaction further confirms that the hydrogen bond between the gas and the membrane surface is formed between the hydroxyl hydrogen bond donor on the SXFA surface and the oxygen atom hydrogen bond acceptor in the DMMP gas. The eight consecutive DMMP detections in Fig. 18 show favorable repeatability of the sensor.

## Conclusions

In this work, a novel passive WSAW gas sensor was successfully developed and used to detect the organophosphorous compound DMMP. The results showed that the WSAW sensor had a stability of less than 31°/h, and it could transmit and receive wireless transmission signals within a range of 0–90 cm. Simultaneously, the surface of the SXFA film was analyzed using a polarizing microscope, SEM, and AFM, and favorable uniformity of the film was confirmed. The detection limit for DMMP at a distance of 20 cm was 0.48 mg/m^3^, and the sensitivity was 4.62°/(mg/m^3^). As a new type of chemical gas sensor technology, WSAW still presents certain limitations, such as the sensor noise increasing with the extension of distance between the transmitting and receiving antennas. This noise makes it difficult to detect low concentration gas. Although the detection signal is not related to distance, it may still be obfuscated by noise, which may be solved by shielding EM interference, increasing radar transmission power and thus reducingthe noise.

## References

[1] Hyunsook J, Dongha K, Kyoung CL (2017). Fate of sulfur mustard on soil: evaporation, degradation, and vapor emission. Environ. Pollut..

[2] George WW, Olga BK (2000). Reactions of VX, GD, and HD with nanosize CaO: autocatalytic dehydrohalogenation of HD. J. Phys. Chem. B..

[3] Singer BC, Hodgson AT, Destaillats H (2005). Indoor sorption of surrogates for sarin and related nerve agents. Environ. Sci. Technol..

[4] Nimal AT (2009). Development of handheld SAW vapor sensors for explosives and CW agents. Sens. Actuators B: Chem..

[5] Matatagui D (2012). Love-wave sensor array to detect, discriminate and classify chemical warfare agent simulants. Sens. Actuators B: Chem..

[6] Matatagui D, Marti J (2011). Chemical warfare agents simulants detec tion with an optimized SAW sensor array. Sens. Actuators B: Chem..

[7] Pan Y (2022). Interface and sensitive character istics of the viscoe- lastic film used in a surface acoustic wave gas sensor. ACS Sens..

[8] Pan Y, Zhang GW (2020). Environmental characteristics of surface acoustic wave devices for sensing organophosphorus vapor. Sens. Actuators B: Chem..

[9] Zheng JP, Zhou J (2020). 30 Ghz surface acoustic wave transducers with extremely high mass sensitivity. Appl. Phys. Lett..

[10] Chen Z (2020). Ultrahigh frequency surface acoustic wave sensors with giant mass-loading effects on electrodes. ACS Sens..

[11] Lamanna L (2020). Conformable surface acoustic wave biosensor for E- coli fabricated on PEN plastic film. Biosens. Bioelectron..

[12] Liu XL, Wang W (2018). Enhanced sensitivity of a hydrogen sulfide sensor based on surface acoustic wave at room temperature. Sensors.

[13] Min L, Kan H, Chen ST (2019). Colloidal quantum dot-based sur face acoustic wave sensors for NO2-sensing behavior. Sens. Actuators B: Chem..

[14] Constantinoiu I, Viespe C (2020). ZnO metal oxide semiconductor in surface acoustic wave sensors: a review. Sensors.

[15] Kim J, Park H, Kim J (2020). SAW chemical array device coated with polymeric sensing materials for the detection of nerve agents. Sensors.

[16] Länge K (2019). Bulk and surface acoustic wave sensor arrays for multi- analyte detection: a review. Sensors.

[17] Minot B (2016). Sensitive materials for chemical agents vapor detection using SAW sensors. Procedia Eng..

[18] Sayago I, Matatagui D (2016). Graphene oxide as sensitive layer in Love-wave surface acoustic wave sensors for the detection of chemical warfare agent simulants. Talanta.

[19] Singh H, Raj VB (2014). Metal oxide SAW E-nose employing PCA and ANN for the identification of binary mixture of DMMP and methanol. Sens. Actuators B: Chem..

[20] Raj VB (2015). Origin and role of elasticity in the enhanced DMMP detection by ZnO/SAW sensor. Sens. Actuators B: Chem..

[21] Pan Y (2014). A SAW-based chemical sensor for detecting sulfur- containing organophosphorus compounds using two-step self- assembly and molecular imprinting technology. Sensors.

[22] Zheng Q (2020). Advances in the chemical sensors for the detec- tion of DMMP — A simulant for nerve agent sarin. Procedia Eng..

[23] Zhu ZX, Chen X, Ma G (2015). Research and application progress of passive sensors. Smart Grid.

[24] Lurz F, Ostertag T (2018). Reader architectures for wireless surface acoustic wave sensors. Sensors.

[25] Wright RF, Lu P (2019). Corrosion sensors for structural health mon itoring of oil and natural gas infrastructure: a review. Sensors.

[26] Pan Y (2021). Wireless passive surface acoustic wave (SAW) tech nology in gas sensing. Sens. Rev..

[27] Pohl A (2000). A review of wireless SAW sensors. IEEE Trans. Ultrason. Ferroelectr. Freq. Control.

[28] Bruno F, Jean MF (2015). High temperature packaging for surface acoustic wave transducers acting as passive wireless sensors. Sens. Actuators A: Phys..

[29] Stelzer A (2008). Wireless sensor marking and tem perature meas- urement with SAW-identification tags. Measurement.

[30] Bruckner G, Bardong J (2019). Wireless readout of multiple SAW temperature sensors. Sensors.

[31] Shu L (2015). High-temperature SAW wireless strain sensor with langasite. Sensors.

[32] Tang ZZ, Wu WY (2015). Water pressure sensing based on wireless passive SAW technology. Procedia Eng..

[33] Nicolay P, Chambon H (2018). A LN/Si-based SAW pressure sensor. Sensors.

[34] Binder A, Bruckner G (2013). Wireless surface acoustic wave pres sure and temperature sensor with unique identification based on LiNbO3. IEEE Sens. J..

[35] Li B, Yassinem O (2015). A surface acoustic wave passive and wire less sensor for magnetic fields, temperature, and humidity. IEEE Sens. J..

[36] Lieberzeit PA, Christian P (2009). SAW RFID-tags for mass- sensitive detection of humidity and vapors. Sensors.

[37] Wang W, Lee K (2007). A novel wireless, passive CO2 sensor incur porating a surface acoustic wave reflective delay line. Smart Mater. Struct..

[38] Wang W (2008). Enhanced sensitivity of wireless chemical sensor based on Love wave mode. Jpn. J. Appl. Phys..

[39] Wang W, Kim C (2009). Wireless surface acoustic wave chemical sensor for simultaneous measurement of CO2 and humidity. J. Micro/Nanolith. Mems Moems..

[40] Ye XS, Fang L (2011). Studies of a high-sensitive surface acoustic wave sensor for passive wireless blood pressure measurement. Sens. Actuators A: Phys..

[41] Zdravko PK, Any IS (1992). Surface acoustic wave gas sensors. Sens. Actuators B: Chem..

[42] Wang YZ, Chyu MK (2014). Passive wireless surface acoustic wave CO2 sensor with carbonnanotube nanocomposite as an interface layer. Sens. Actuators A: Phys..

[43] Devkota J (2018). Zeolitic imidazole framework-coated acoustic sensors for room temperature detection of carbon dioxide and me- thane. Nanoscale.

[44] Lim C, Wang W (2011). Development of SAW-based multigas sen sor for simultaneous detection of CO2 and NO2. Sens. Ac- tuators B: Chem..

[45] Rana L (2018). Fabrication of surface acoustic wave based wireless NO2 gas sensor. Surf. Coat. Technol..

[46] Guo XW (2017). Preparation of LC wireless passive gas sensor and study on the gas sensing properties of ammonia gas. Chin. J. Sens. Actuators.

[47] Greve DW, Zheng P (2013). Surface acoustic wave devices for harsh environment wireless sensing. Sensors.

[48] Shu L, Xia YD (2019). The investigation of a SAW oxygen gas sen- sor operated at room temperature, based on nanostructured ZnxFeyO films. Sensors.

[49] Xu FQ (2015). Development of a wireless and passive SAW-based chemical sensor for organophosphorous compound detection. Sensors.

[50] Wang Y (2008). A passive wireless temperature sensor for harsh environment applications. Sensors.

[51] Reindl L, Shrena IM (2004). Wireless measurement of temperature using surface acoustic wave sensors.. IEEE Trans. Ultrason. Ferroelectr. Freq. Control..

[52] Wang W, Lee KK (2007). Optimal design on SAW sensor for wire- less pressure measurement based on reflective delay line. Sens. Actuators A: Phys..

[53] Pan Y (2022). Development of a SAW poly(epichlorohydrin) gas sensor for detection of harmful chemicals. Anal. Methods.

